# Mutation of *ONAC096* Enhances Grain Yield by Increasing Panicle Number and Delaying Leaf Senescence during Grain Filling in Rice

**DOI:** 10.3390/ijms20205241

**Published:** 2019-10-22

**Authors:** Kiyoon Kang, Yejin Shim, Eunji Gi, Gynheung An, Nam-Chon Paek

**Affiliations:** 1 Department of Plant Science, Plant Genomics and Breeding Institute, Research Institute of Agriculture and Life Sciences, Seoul National University, Seoul 08826, Korea; kykang7408@snu.ac.kr (K.K.); yejin5648@snu.ac.kr (Y.S.); abc1209abc@snu.ac.kr (E.G.); 2 Department of Plant Molecular Systems Biotechnology, Crop Biotech Institute, Kyung Hee University, Yongin 17104, Korea; genean@khu.ac.kr

**Keywords:** rice, grain yield, tillering, leaf senescence, abscisic acid (ABA), NAM/ATAF1/2/CUC2 (NAC)

## Abstract

Exploring genetic methods to improve yield in grain crops such as rice (*Oryza sativa*) is essential to help meet the needs of the increasing population. Here, we report that rice ONAC096 affects grain yield by regulating leaf senescence and panicle number. *ONAC096* expression increased rapidly in rice leaves upon the initiation of aging- and dark-induced senescence. Two independent T-DNA insertion mutants (*onac096-1* and *onac096-2*) with downregulated *ONAC096* expression retained their green leaf color during natural senescence in the field, thus extending their photosynthetic capacity. Reverse-transcription quantitative PCR analysis showed that *ONAC096* upregulated genes controlling chlorophyll degradation and leaf senescence. Repressed *OsCKX2* (encoding cytokinin oxidase/dehydrogenase) expression in the *onac096* mutants led to a 15% increase in panicle number without affecting grain weight or fertility. *ONAC096* mediates abscisic acid (ABA)-induced leaf senescence by upregulating the ABA signaling genes *ABA INSENSITIVE5* and *ENHANCED EM LEVEL*. The *onac096* mutants showed a 16% increase in grain yield, highlighting the potential for using this gene to increase grain production.

## 1. Introduction

Rice (*Oryza sativa*) is a major staple crop that feeds over one-third of the worldwide population [1]. Cultivated rice has been genetically improved to increase grain yields [2,3,4]. Rice grain yield is mainly determined by the number of panicles per plant, grains per panicle, and weight of each grain [5]. Among these traits, one key factor in determining panicle number is tillering, which is coordinately regulated by many factors [6,7]. For example, rice *TEOSINTE BRANCHED1* (*OsTB1*) encodes a basic helix–loop–helix transcription factor, and the *OsTB1* loss-of-function mutation *fine culm1* (*fc1*) increases tiller number [8].

Leaf senescence, the final stage of leaf development, is affected by complex regulatory networks [9]. The molecular mechanisms controlling the onset and progression of leaf senescence in rice are well understood. Mutants of rice *nonyellow coloring3* (*NYC3*), encoding a plastid-localizing α/β hydrolase, exhibit a stay-green phenotype during dark-induced senescence [10]. Knockdown of rice *pheophorbide a oxygenase* (*OsPAO*) and *red chlorophyll catabolite1* (*OsRCCR1*) prolongs leaf greenness during dark incubation [11]. The delayed leaf yellowing phenotype of *stay-green* (*sgr*) mutants under dark-induced and natural senescence conditions is due to a lack of magnesium-dechelating activity [12,13].

In addition, several senescence-induced transcription factors (TFs) regulate the expression of senescence-associated genes (SAGs) by directly or indirectly binding to the *cis* elements in their promoter regions. Rice NAC2 (OsNAC2), a member of the NAC (NAM, ATAF1, and CUC2) TF family, promotes leaf senescence by upregulating the expression of genes associated with chlorophyll degradation and leaf senescence [14]. Overexpression of *OsNAP* (rice *NAC-*like, activated by *apetala3/pistillata*) in the gain-of-function mutant *prematurely senile 1-D* (*ps1-D*) results in premature natural and dark-induced leaf senescence [15]. OsNAP directly binds to the promoters of *SGR*, *OsRCCR1*, *NYC3*, and *Osl57*. The expression of *OsNAC5* and *OsNAC*6 is upregulated during leaf senescence [16,17]. Delayed leaf senescence can extend grain filling, thereby increasing crop yields. For instance, plants with downregulated *OsNAP* and *OsNAC2* expression via RNAi exhibited a stay-green phenotype and increased grain yields [14,15].

The plant hormones auxin and cytokinin play key roles in regulating tillering. Membrane-localized auxin transporter proteins help determine auxin distribution within plant tissues, thus affecting organogenesis and morphogenesis [18]. The rice *PIN-FORMED* (*OsPIN*) genes encode auxin efflux transporters involved in rice tillering. Indeed, transgenic plants overexpressing *OsPIN2* produce more tillers bearing grain (effective tillers), leading to increased grain yield [19]. The suppression of *OsPIN5b* by RNA interference (RNAi) improves grain yield by increasing tiller number, panicle length, and total number of seeds [20]. The rice TRANSPORT INHIBITOR RESPONSE1 (OsTIR1) regulates tillering by repressing the auxin transporter gene *OsAUX1* [21]. In addition, cytokinin oxidase/dehydrogenase (CKX), which irreversibly degrades cytokinin, regulates rice tillering; the downregulation of *OsCKX2* leads to enhanced tillering and thus higher productivity in rice [22].

Abscisic acid (ABA) is a pivotal phytohormone that regulates multiple developmental processes, including leaf senescence, seed maturation and dormancy, and organ abscission [23,24,25]. Endogenous ABA contents increase during leaf senescence in various plant species such as tobacco (*Nicotiana rustica* L.) [26], oat (*Avena sativa*) [27], rice [28], maize (*Zea mays*) [29], and *Arabidopsis thaliana* [30]. ABA promotes leaf senescence by inducing the expression of SAGs such as *SGR*, *OsRCCR1*, *Osh36*, and *Osl57* [15]. Genes related to ABA biosynthesis and signaling are upregulated in senescing rice and *Arabidopsis* leaves [31,32]. Exogenous ABA treatment induces the expression of NAC TF genes, including *VND-INTERACTING2* (*VNI2*) [33], *ORESARA1* [34], *OsNAP* [15], and *OsNAC2* [14].

Previous genome-wide analysis demonstrated that *ONAC096* is upregulated in rice seedlings under ABA treatment and abiotic stresses such as salinity, drought, and cold stress [35]. However, the molecular mechanisms underlying the role of *ONAC096* in regulating tillering and leaf senescence in rice are poorly understood. Here, we uncovered possible roles of *ONAC096* in rice tillering and leaf senescence. Our findings suggest that downregulating *ONAC096* increases tiller number by reducing *OsCKX2* expression and delays leaf senescence by reducing the expression of chlorophyll degradation genes (CDGs) and SAGs. These processes, which occur in the *onac096* mutant, increase panicle number and photosynthetic activity, leading to higher grain yields. Thus, regulating the expression of *ONAC096* could help improve crop productivity.

## 2. Results

### 2.1. Characterization of ONAC096

Among the rice NAC proteins (ONACs) encoded by the 140 ONAC genes in the rice genome [36], a few ONACs are known to function in leaf senescence (OsNAP [15], ONAC106 [37], OsNAC2/ONAC004 [14], and ONAC011 [38]). To further explore the roles of ONACs in regulating leaf senescence, we investigated the phylogenetic relationships between ONACs and seven *Arabidopsis* NAC proteins (ANACs) whose regulatory roles in leaf senescence have been determined (Figure S1). This phylogenetic analysis revealed that six ONACs, ONAC022 (Os03g04070), ONAC063 (Os08g33910), ONAC066 (Os03g56580), ONAC095 (Os06g51070), ONAC096 (Os07g04560), and ONAC140 (Os12g43530), were closely clustered with JUNGBRUNNEN1 (JUB1). 

According to the public expression data from GENEVESTIGATOR (https://genevestigator.com/gv/) and RiceXPro (http://ricexpro.dna.affrc.go.jp/), while the expression of *ONAC063* drops sharply at the reproductive stage, *ONAC022*, *ONAC066*, *ONAC095*, *ONAC096*, and *ONAC140* expression gradually increases during natural senescence in the field (Figure S2). Among the six *ONAC* candidates, we examined the potential roles of *ONAC096* in the onset and progression of leaf senescence in detail. *ONAC096* comprises 1904 nucleotides, with a 1032 bp open reading frame encoding a protein made of 343 amino acids. Amino acid sequence alignments between *ONAC096* and its putative orthologs indicated that the NO APICAL MERISTEM (NAM) domain was highly conserved among diverse plant species (Figure S3).

### 2.2. ONAC096 is Upregulated During Leaf Senescence 

**Figure 1:**
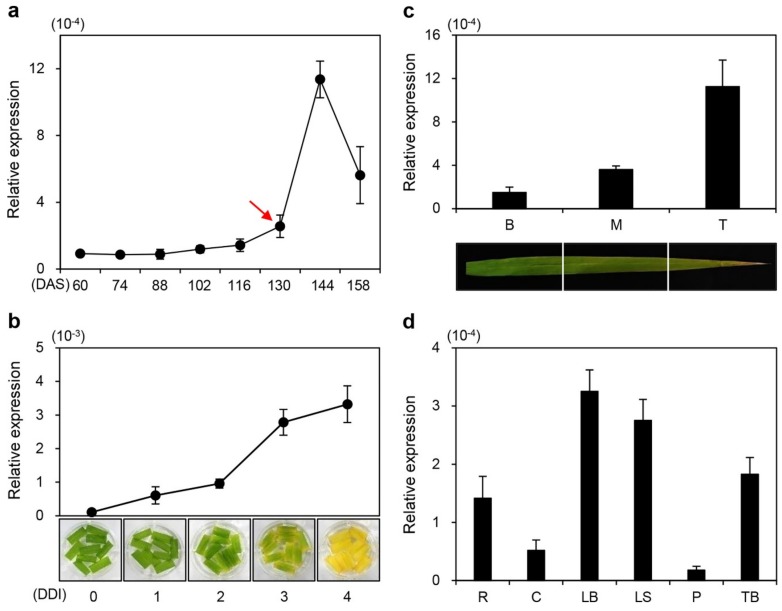
Expression profiles of *ONAC096*. (**a**–**c**) *ONAC096* transcript levels were measured in the leaves of wild-type (WT) plants grown in the field under natural long-day (NLD) conditions (≥14 h light per day) (**a,c**) or in detached leaves of WT plants grown in a growth chamber for 3 weeks under long-day (LD) conditions (14 h light/10 h dark) (**b**). (**a**) *ONAC096* is strongly expressed in WT flag leaves after heading. The red arrow indicates the heading date (130 days after seeding (DAS)). (**b**) *ONAC096* expression gradually increases in detached leaves of 4-week-old WT plants in which senescence was induced in complete darkness in 3 mM MES buffer (pH 5.8) at 28 °C. (**c**) *ONAC096* transcript levels in senescing WT flag leaves that were divided into three regions at the ripening stage (158 DAS). B, bottom; M, middle; T, tip. (**d**) *ONAC096* is differentially expressed in WT tissues separated from the tiller base (TB) at the tillering stage (88 DAS) in root (R), culm (C), leaf blade (LB), leaf sheath (LS), and panicle (P) tissue at the heading stage (130 DAS). *ONAC096* transcript levels were determined by RT-qPCR and normalized to that of *OsUBQ5*. Relative expression was calculated using the ΔΔC_T_ method. Mean and standard deviations were obtained from five (**a,c,d**) and three biological repeats (**b**). The experiment was repeated twice with similar results.

To investigate whether senescence affected the expression of *ONAC096*, we monitored the changes in *ONAC096* transcript levels in the flag leaves of wild-type (WT; *japonica* cultivar ‘Dongjin’) plants grown under natural long-day conditions (≥14 h light per day) in the field (37°N latitude, Suwon, South Korea) via reverse-transcription quantitative PCR (RT-qPCR). *ONAC096* was sharply upregulated in flag leaves at 144 days after seeding (DAS) (Figure 1a) and in detached leaves during dark-induced senescence (DIS) (Figure 1b). 

Higher *ONAC096* transcript levels were detected in the tip (T) region than in the middle (M) and bottom (B) regions of senescing flag leaves (Figure 1c). We examined the spatial expression patterns of *ONAC096* in rice organs at the tillering and heading stages. These detached rice organs included the tiller base at the tillering stage (88 DAS) and the leaf blade, leaf sheath, root, culm, and panicle at the heading stage (130 DAS) (Figure 1d). RT-qPCR analysis demonstrated that *ONAC096* was preferentially expressed in tissues of the leaf blade, leaf sheath, and tiller base, suggesting that ONAC096 might regulate leaf senescence and tillering in rice.

### 2.3. The onac096 Mutation Delays Leaf Yellowing during Natural and Dark-Induced Senescence 

To determine the biological functions of *ONAC096* in leaf senescence, we obtained two T-DNA insertion mutants (PFG_1B-02928: *onac096-1* and PFG_3A-08770: *onac096-2*) from the RiceGE database (http://signal.salk.edu/cgi-bin/RiceGE), in which a T-DNA fragment was independently integrated into intron 1 (*onac096-1*) or intron 2 (*onac096-2*) of *ONAC096*. To determine whether these two mutant lines were knockout or knockdown mutants, we compared the transcript levels of *ONAC096* in the leaves of 2-month-old WT, *onac096-1*, and *onac096-2* (hereafter, *onac096* mutants) plants. RT-qPCR analysis showed that *ONAC096* transcript levels were significantly reduced in the *onac096* mutants compared to the WT, indicating that both lines were knockdown mutants (Figure S4). 

**Figure 2:**
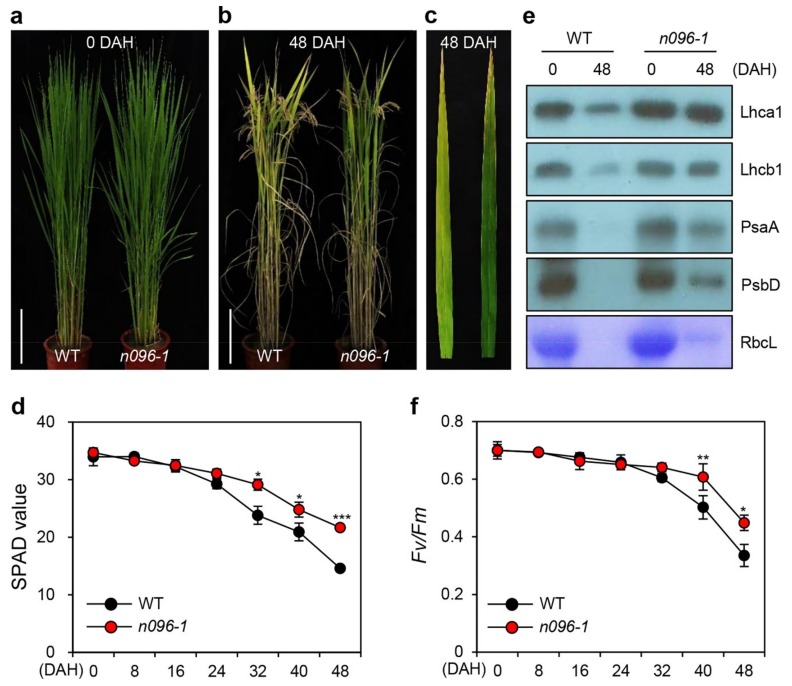
The *onac096-1* mutant exhibits delayed natural leaf senescence. The rice plants were grown in the field under natural long-day conditions (≥14 h light/d). (**a**–**c**) Phenotypes of WT and *onac096-1* (*n096-1*) plants at 0 DAH (**a**) and 48 DAH (**b**). Bars = 20 cm. (**c**) Senescing flag leaves of WT (left) and *onac096-1* (right) plants at 48 DAH. The photographs are representative of five independent plants. (**d**–**f**) Changes in photosynthetic proteins (**d**), SPAD values (**e**), and PSII activity (*Fv/Fm*) (**f**) in flag leaves of WT and *onac096-1* plants after heading. SPAD values (**d**) and *Fv/Fm* ratios (**f**) were measured in the middle part of a leaf blade every 8 d from 0 to 48 DAH. (**e**) Immunoblotting performed using antibodies against photosynthetic proteins (Lhca1, Lhcb1, PsaA, and PsbD). Mean and standard deviations were obtained from 10 biological repeats (**d,f**). Asterisks indicate statistically significant differences between *onac096-1* and WT, as determined by Student’s *t*-test (*p < 0.05, **p < 0.01, and ***p < 0.001). The experiment was repeated twice with similar results.

There was no obvious phenotypic difference in leaf greenness between the WT and *onac096-1* until the heading stage (Figure 2a). However, at 48 days after heading (DAH), *onac096-1* showed delayed leaf yellowing compared to the WT (Figure 2b,c). Consistent with the persistence of leaf greenness, the SPAD value, a parameter measuring leaf greenness, indicated that there were higher levels of green pigments in the flag leaves of *onac096-1* after 32 DAH versus the WT (Figure 2d). 

We then examined the levels of photosynthetic proteins by immunoblotting with antibodies against photosystem II (PSII) proteins (antenna: Lhcb1 and core: PsbD) and photosystem I (PSI) proteins (antenna: Lhca1 and core: PsaA) in the flag leaves of WT and *onac096-1* at 48 DAH, revealing that more photosynthetic proteins remained in *onac096-1* (Figure 2e). We measured the *Fv/Fm* ratio (efficiency of PSII) in the flag leaves of the WT and *onac096-1* after heading. Compared to the WT, *onac096-1* maintained higher photosynthetic activity 40 DAH (Figure 2f). The retained leaf greenness of rice *onac096* mutants improved photosynthetic capacity during grain filling, indicating it is a functional stay-green mutant [39]. 

**Figure 3:**
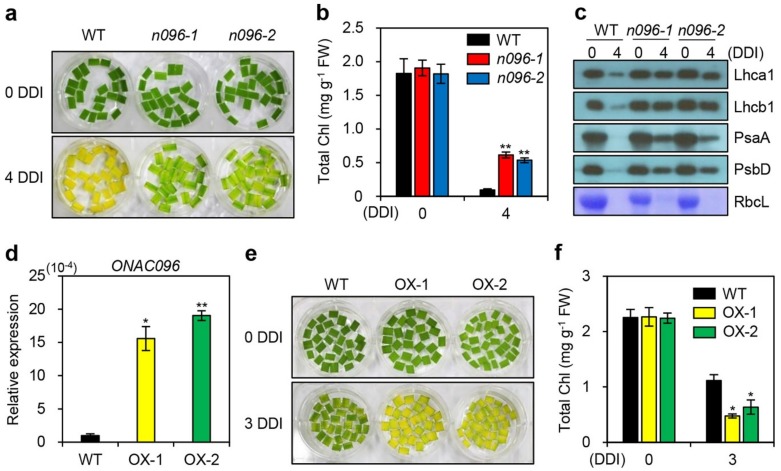
*ONAC096* accelerates leaf yellowing during dark-induced senescence. (**a**–**c,e,f**) Detached leaves of 3-week-old plants were incubated in 3 mM MES buffer (pH 5.8) in complete darkness at 28 °C. Leaf yellowing (**a**) and total chlorophyll contents (**b**) were monitored in *onac096* at 0 and 4 days of dark incubation (DDI). (**c**) Immunoblotting of detached leaves from WT and *onac096* plants at 0 and 4 DDI using antibodies against photosynthetic proteins (Lhca1, Lhcb1, PsaA, and PsbD). (**d**) Expression of *ONAC096* measured in the leaves of *ONAC096*-OX plants (OX-1 and OX-2) grown in a growth chamber for 3 weeks under long-day conditions. *ONAC096* transcript levels were measured by RT-qPCR and normalized to that of *OsUBQ5*. Relative expression was calculated using the ΔΔC_T_ method. (**e,f**) The detached leaves of *ONAC096*-OX plants exhibited accelerated leaf yellowing (**e**) and reduced total chlorophyll levels (**f**) at 3 DDI. (**b,d,f**) Mean and standard deviations were obtained from three biological repeats. Asterisks indicate statistically significant differences between *onac096* (**b**) and *ONAC096*-OX plants (**d,f**) compared to the WT at 4 and 3 DDI, respectively, according to Student’s *t*-test (*p < 0.05 and **p < 0.01). The experiments were repeated twice with similar results. Chl, chlorophyll; FW, fresh weight.

To identify whether leaf yellowing was delayed in the *onac096* mutants during dark-induced senescence, we detached the leaves of 3-week-old WT and *onac096* plants and floated them on 3 mM 2-(*N*-morpholino)ethanesulfonic (MES) buffer (pH 5.8) in complete darkness at 28 °C. The mutant leaves retained their green color longer than WT leaves at 4 days of dark incubation (DDI) (Figure 3a), indicating that *onac096* leaves had higher total chlorophyll contents than WT leaves (Figure 3b). The levels of photosynthetic proteins (Lhca1, Lhcb1, PsaA, and PsbD) also remained higher in detached leaves of *onac096* versus the WT (Figure 3c).

To further confirm the regulatory role of ONAC096 in leaf senescence, we generated two independent transgenic rice lines overexpressing *ONAC096* (*ONAC096*-OX; OX-1 and OX-2) (Figure 3d). The detached leaves of 3-week-old *ONAC096*-OX plants exhibited accelerated leaf yellowing compared to the WT under dark-induced senescence (Figure 3e). Consistent with this observation, total chlorophyll contents were significantly reduced in *ONAC096*-OX versus WT leaves at 3 DDI (Figure 3f), indicating that the overexpression of *ONAC096* contributed to premature leaf senescence. These findings indicate that ONAC096 promotes the onset and progression of leaf senescence in rice.

### 2.4. Mutation of ONAC096 Improves Grain Yield by Increasing the Number of Panicles Per Plant

**Figure 4:**
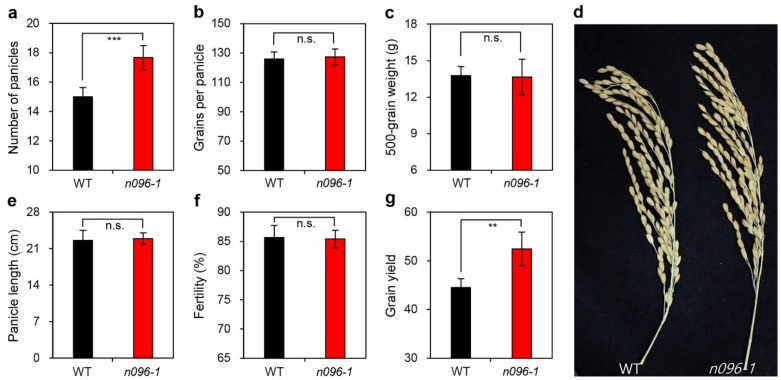
Downregulating *ONAC096* increases grain yield. Agronomic traits of WT and *onac096-1* (*n096-1*) plants were investigated after harvest in the autumn. Comparison of the number of panicles (**a**), grains per panicle (**b**), 500-grain weight (**c**), phenotype of panicles (**d**), panicle length (**e**), fertility (**f**), and grain yield (**g**) between the WT and *onac096-1*. Mean and standard deviations were obtained from 10 measurements. Asterisks indicate significant differences between WT and *onac096-1* according to Student *t*-test (**p < 0.01 and ***p < 0.001; n.s., not significant).

In addition to the functional stay-green trait, to identify whether the mutation of *ONAC096* also affected grain yield and yield components in rice, we investigated several agronomic traits in the *onac096-1* mutant grown in the paddy field under natural long-day (NLD) conditions. These agronomic traits included number of panicles per plant, number of spikelets per panicle, 500-grain weight, main panicle length, grain yield per plant, and spikelet fertility. Significantly more panicles were present in *onac096-1* versus WT plants (Figure 4a). However, WT and *onac096-1* plants had similar numbers of spikelets per panicle, 500-grain weight, main panicle length, grain yield per plant, and spikelet fertility (Figure 4b–f). Thus, the increase in number of panicles per plant in *onac096-1*, which resulted in 16% greater grain yields, occurred independently of the delayed senescence trait (Figure 4g).

**Figure 5:**
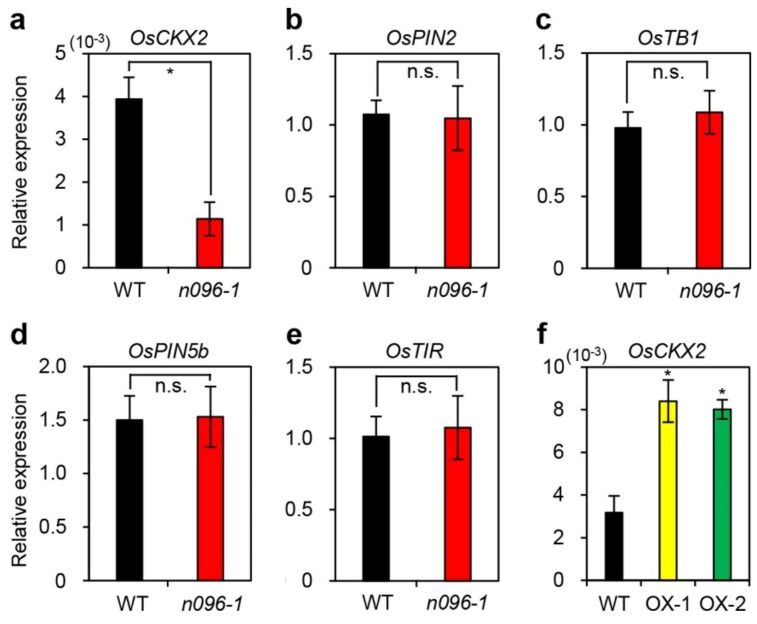
ONAC096 positively regulates *OsCKX2* expression. Total RNA was isolated from the tiller bases of plants at the tillering stage. *OsCKX2* (**a**), *OsPIN2* (**b**), *OsTB1* (**c**), *OsPIN5b* (**d**), and *OsTIR* (**e**) transcript levels were compared between the WT and *onac096-1* (*n096-1*). (**f**) Overexpression of *ONAC096* upregulates the expression of *OsCKX2*. The transcript levels of tillering-related genes were measured by RT-qPCR and normalized to that of *OsUBQ5*. Relative expression was calculated using the ΔΔC_T_ method. Mean and SD values were obtained from three biological repeats. Asterisks indicate statistically significant differences between *onac096-1* (**a**–**e**) and *ONAC096*-OX plants (OX-1 and OX-2) (**f**) compared to the WT, as determined by Student’s *t*-test (*p < 0.05; n.s., not significant). The experiments were repeated twice with similar results.

Tillering is an important agronomic trait that largely determines the number of panicles per plant in rice [40,41]. Several genetic regulators of rice tillering have been isolated and characterized, including *OsCKX2* [22], *OsPIN2* [19], *OsTB1* [8], *OsPIN5b* [20], and *OsTIR1*, [21]. Thus, we examined the expression levels of these genes in the tiller bases of field-grown WT and *onac096-1* plants at the tillering stage (Figure 5a–e) via RT-qPCR. Among these five genes, *OsCKX2* was significantly downregulated in the tiller bases of *onac096-1* plants but was highly expressed in those of *ONAC096*-OX plants (Figure 5f). These results suggest that downregulating *ONAC096* enhanced tillering activity, possibly by decreasing *OsCKX2* expression, thereby leading to an increase in the number of panicles per plant

### 2.5. ONAC096 Upregulates CDG and SAG Expression During Leaf Senescence

**Figure 6:**
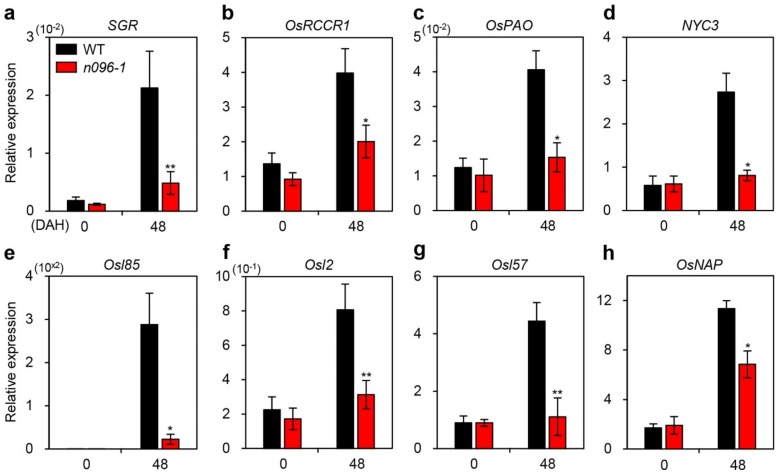
Altered expression of chlorophyll degradation genes (CDGs) and senescence-associated genes (SAGs) in *onac096-1* during natural senescence. Total RNA was isolated from flag leaves of the WT and *onac096-1* (*n096-1*) at 0 and 48 DAH. The transcript levels of CDGs (**a**–**d**) and SAGs (**e**–**h**) were determined by RT-qPCR and normalized to that of *OsUBQ5*. Relative expression was calculated using the ΔΔC_T_ method. Mean and SD values were obtained from three biological repeats. Asterisks indicate statistically significant differences between *onac096-1* and the WT at 48 DAH according to Student’s *t*-test (*p < 0.05 and **p < 0.01). The experiments were repeated twice with similar results.

Leaf senescence involves a series of events regulated by CDGs and SAGs. These genes encode chlorophyll catabolic enzymes (SGR [12]; OsRCCR1 and OsPAO [11]; NYC3 [10]) and proteins involved in amino acid metabolism (Osl2), fatty acid metabolism (Osl85 and Osl57) [42], and a senescence-associated NAC transcription factor (OsNAP) [15]. We evaluated the transcript levels of CDGs and SAGs in flag leaves during natural senescence and in detached leaves of WT and *onac096* plants during dark-induced senescence. The transcript levels of CDGs and SAGs were significantly reduced in *onac096* at 48 DAH and 4 DDI compared to the WT (Figure 6; Figure S5a–h). To confirm this finding, we measured the transcript levels of CDGs and SAGs in the leaves of 3-week-old WT and *ONAC096*-OX plants (Figure S5i–p). The CDGs and SAGs were upregulated in *ONAC096*-OX plants. Together, these findings suggest that ONAC096 positively regulates chlorophyll degradation and SAG expression during leaf senescence.

### 2.6. ONAC096 Mediates ABA-Induced Leaf Senescence 

**Figure 7:**
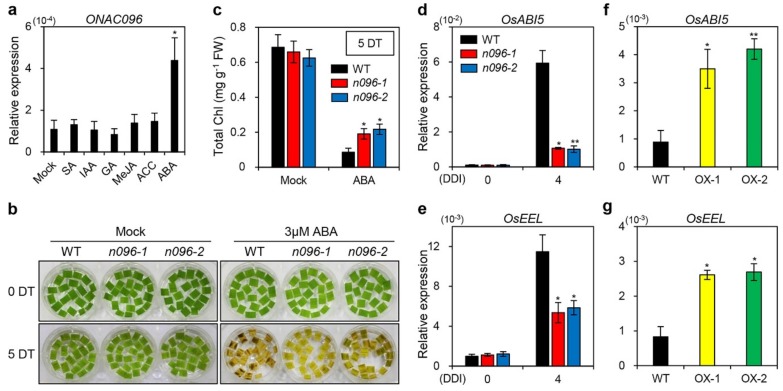
Abscisic acid (ABA) hyposensitivity of *onac096* mutants. (**a**) WT seedlings were grown in MS Phytoagar medium for 10 d under continuous light conditions at 28 °C, followed by incubation in MS liquid medium supplemented with 100 µM SA, 100 µM IAA, 100 µM GA, 100 µM MeJA, 10 mM ACC, or 100 µM ABA. Seedlings incubated in 0.5× MS liquid medium without phytohormones were used as a mock control. Total RNA was isolated from the leaves at 12 h after treatment. Asterisks indicate statistically significant differences between ABA treatment and the mock control, as determined by Student’s *t*-test (*p < 0.05). (**b,c**) Detached leaves of 3-week-old WT and *onac096* plants (*n096-1* and *n096-2*) were treated with 3 mM 3 mM MES buffer (pH 5.8) containing 3 µM ABA under continuous light conditions at 28 °C. Detached leaves incubated in 3 mM MES buffer (pH 5.8) without ABA were used as a mock control. (**b**) The ABA hyposensitive phenotype was observed at 0 and 5 days of treatment (DT). (**c**) Total chlorophyll contents in detached leaves of WT and *onac096* plants were measured at 5 DT. (**d**–**g**) Total RNA was isolated from detached leaves of 3-week-old WT and *onac096* plants under dark-induced senescence (**d,e**) or attached leaves of WT and *ONAC096*-OX plants grown in paddy soil for 3 weeks under LD conditions (**f,g**). (**a,d**–**g**) *ONAC096* (**a**), *OsABI5* (**d,f**), and *OsEEL* (**e,g**) transcript levels were measured by RT-qPCR and normalized to that of *OsUBQ5*. Relative expression was calculated using the ΔΔC_T_ method. Mean and SD values were obtained from three biological repeats. Asterisks indicate statistically significant differences between *onac096* and *ONAC096*-OX plants compared to the WT, as determined by Student’s *t*-test (*p < 0.05 and **p < 0.01). The experiments were repeated twice with similar results. FW, fresh weight.

ABA is a phytohormone that promotes leaf senescence by activating senescence-associated regulatory pathways [9,43]. To investigate whether ABA affects the expression of *ONAC096*, we examined *ONAC096* transcript levels in 10-day-old WT seedlings treated with salicylic acid (SA), indole-3-acetic acid (IAA), gibberellin acid (GA), methyl-jasmonic acid (MeJA), 1-aminocyclo-propane-1-carboxylic acid (ACC), or ABA via RT-qPCR. *ONAC096* was exclusively upregulated in response to ABA treatment (Figure 7a), suggesting that *ONAC096* is involved in ABA-dependent senescence induction pathways. To confirm this notion, we observed leaf yellowing in the detached leaves of WT and *onac096* plants incubated in 3 mM MES buffer (pH 5.8) containing 3 µM ABA under continuous light conditions. The *onac096* leaves retained more green coloration than WT leaves at 5 d of dark treatment (Figure 7b) because of higher total chlorophyll contents in *onac096* (Figure 7c). 

The expression of ABA signaling genes *ABA INSENSITIVE5* (*ABI5*) [44] and *ENHANCED EM LEVEL* (*EEL*) [45], encoding bZIP transcription factors, is induced by leaf senescence [46]. We, therefore, measured the expression levels of ABA signaling genes in the detached leaves of WT and *onac096* plants under dark-induced senescence. The mutation of *ONAC096* reduced the expression of *ABI5* and *EEL* at 4 DDI (Figure 7d,e). By contrast, the ABA signaling genes were upregulated in the leaves of 3-week-old *ONAC096*-OX plants compared to the WT (Figure 7f,g). Taken together, these findings indicate that ONAC096 upregulated ABA signaling genes, thereby promoting leaf senescence.

## 3. Discussion

### 3.1. Effects of ONAC096 on Leaf Senescence and Tillering

We demonstrated that downregulating *ONAC096* increases tiller number and delays leaf senescence, resulting in improved grain yield in rice. Genes or QTLs affecting the functional stay-green trait could potentially be used to increase production in cereal crops [47,48,49,50,51,52,53]. Rice RNAi lines with reduced transcript levels of NAC TF genes *OsNAP* [15] and *OsNAC2* [14] exhibit a typical functional stay-green phenotype or delayed leaf yellowing due to extended photosynthetic capacity, as well as increased seed-setting rate and 1000-grain weight, leading to higher grain yields [54]. However, the functional stay-green trait is not always beneficial for grain yield and yield components, perhaps because of the unexpected negative effects of senescence-associated genes on plant growth and development, such as growth retardation and reduced seed fertility and grain filling rate. For instance, we previously determined that although rice *coronatine insensitive 1b* (*oscoi1b*) mutants retain high levels of photosynthetic activity and high rates of CO_2_ exchange during grain filling under natural senescence conditions in the field, impaired jasmonate signal transduction in these mutants leads to poor spikelet development, resulting in lower grain yields [46,55]. Furthermore, rice plants overexpressing *DNA-BINDING ONE ZINC FINGER 24* (*OsDOF24*) exhibit delayed leaf yellowing along with prolonged photosynthetic capacity but show shorter plant height and panicle length, fewer spikelets per panicle, and lower seed fertility than the WT, resulting in greatly reduced grain yields [56]. 

To increase grain yields in cereal crops, photosynthates produced in green leaves (source organ) must be efficiently translocated to developing seeds (sink organ) [57]. Therefore, panicle number, spikelet fertility, and grain filling rate are crucial yield components that can increase total grain yield per plant in rice. Here, we determined that *onac096* plants produced more panicles than the WT without any negative effects on spikelet number per panicle, grain weight, panicle length, or seed fertility (Figure 4). In addition, the flag leaves of *onac096* plants showed an extended period of photosynthetic capacity during grain filling (Figure 2f), allowing them to accumulate sufficient amounts of photosynthates to produce increased numbers of spikelets, leading to higher grain yields compared to the WT (Figure 4g).

**Figure 8:**
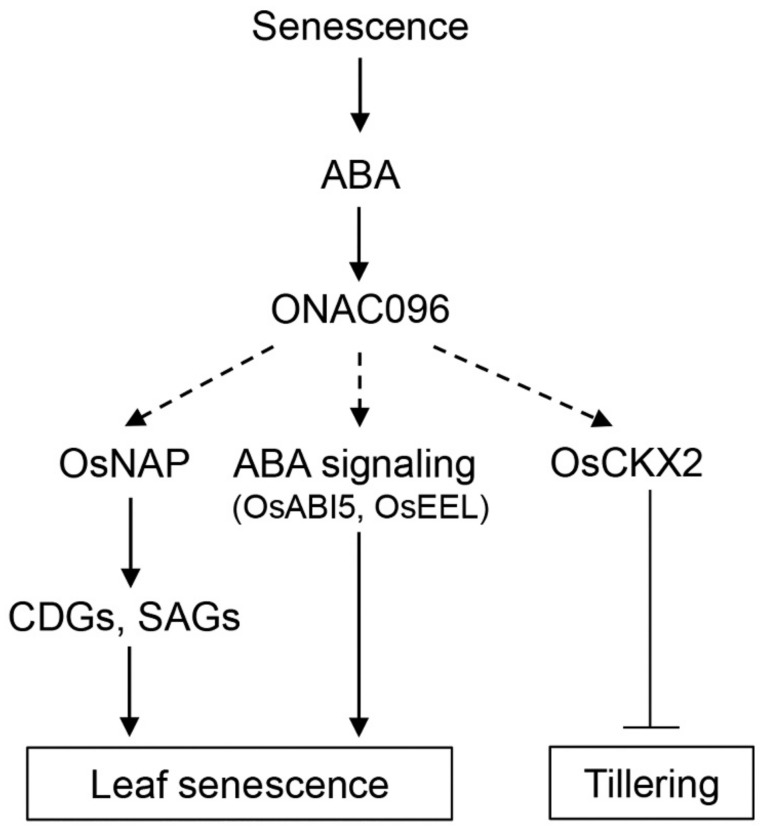
Proposed model of the role of *ONAC096* in leaf senescence and tillering in rice. Arrows indicate upregulation, and lines ending with bars represent downregulation. Solid and dashed lines represent direct and indirect regulation of downstream genes, respectively.

High tillering capacity is a beneficial trait for grain production of rice, since the number of tillers per plant is closely related to the number of panicles per plant [58,59]. The genes controlling tillering in rice encode proteins involved in phytohormone-associated regulatory mechanisms. Auxin distribution (mediated by membrane-localized auxin carriers, including AUX1, PIN, and ABC transporters) determines plant architecture, thereby affecting grain yield components such as tiller number and angle, as well as panicle morphology [59,60,61,62]. Among these regulators, overexpressing *OsPIN5b*, encoding an endoplasmic reticulum (ER)-localized protein, increases endogenous free indole-3-acetic acid (IAA) levels in leaves, roots, and panicles. These altered IAA levels affect auxin-dependent rice architecture, reducing plant height, seed-setting rate, panicle length, and tiller number in rice [20]. The increased tiller angle and number in *OsPIN2*-overexpressing transgenic plants is due to enhanced IAA transport from shoots to roots [19]. Downregulating the auxin receptor gene *OsTIR1* reduced auxin accumulation in auxiliary buds, resulting in increased tillering [21]. Cytokinin oxidase/dehydrogenase 2 (OsCKX2), which functions in cytokinin degradation, also regulates grain production in rice [22,63]. Suppressing *OsCKX2* expression led to increased grain and tiller number and high fertility. The finding that *ONAC096* is expressed in the tiller base suggests that ONAC096 acts as transcriptional regulator of rice tillering (Figure 1d). Indeed, *OsCKX2* transcript levels were significantly lower in *onac096* compared to the WT (Figure 5a), indicating that ONAC096 is involved in *OsCKX2*-mediated rice tillering (Figure 8). 

### 3.2. Involvement of ONAC096 in ABA-Induced Leaf Senescence

Leaf senescence generally occurs in an age-dependent manner and is affected by internal factors such as phytohormones [9,64]. Cytokinin has long been known to inhibit leaf senescence in plants [65,66,67,68]. *OsCKX2* is strongly expressed in rice leaves [63] and is upregulated in shoots in response to exogenous cytokinin treatment [69]. Furthermore, downregulating *OsCKX2* results in increased endogenous cytokinin levels [70]. Based on the finding that *OsCKX2* transcripts strongly accumulate in *ONAC096*-overexpressing transgenic rice plants (Figure 5f), it is likely that ONAC096 promotes leaf senescence by reducing cytokinin levels via effects on *OsCKX2* expression. 

In contrast to the role of cytokinin in leaf senescence, ABA accelerates the onset and progress of leaf senescence [24]. In *Arabidopsis*, the bZIP TFs ABA INSENSITIVE5 (ABI5) and ENHANCED EM LEVEL (EEL) bind to the promoters of *NYE1/SGR1* and *NYC1* to accelerate chlorophyll degradation [71]. Their rice orthologs, *OsABI5* and *OsEEL*, are induced by leaf senescence [46]. In this study, we determined that *ONAC096* was upregulated in response to ABA treatment (Figure 7a) and that *OsABI5* and *OsEEL* transcript levels were significantly lower in *onac096* versus WT plants during dark-induced leaf senescence (Figure 7d,e). These findings indicate that ONAC096 mediates ABA-induced leaf senescence by activating ABA signaling (Figure 8). 

## 4. Materials and Methods

### 4.1. Plant Materials, Growth Conditions, and Experimental Treatments

The rice (*Oryza sativa* ssp. *japonica*) cultivar ‘Dongjin’ and two independent T-DNA insertion mutants (*onac096-1*, PFG_1B-02928 and *onac096-2*, PFG_3A-08770) were cultivated in a rice paddy field under NLD conditions (≥14 h sunlight per day, 37°N latitude) in Suwon, Republic of Korea. The germinated rice seedlings were also grown in a growth chamber under LD conditions (14 h light at 28 °C/10 h dark at 25 °C, 37°N latitude) in Suwon, Republic of Korea. The T-DNA insertion mutants were obtained from the Crop Biotech Institute at Kyung Hee University, Republic of Korea [72]. 

For dark treatment, detached leaves of plants grown in a growth chamber for 3 weeks were incubated in 3 mM 2-(*N*-morpholino)ethanesulfonic (MES) buffer (pH 5.8) with the abaxial side up at 28 °C in complete darkness. For phytohormone treatments, WT seeds were sterilized in 70% ethanol and 2% NaClO and washed three times with sterile water. The sterilized seeds were germinated and grown on half-strength Murashige and Skoog (0.5× MS, Duchefa, Haarlem, The Netherlands) solid medium in a growth chamber under continuous light (90 µmol m^−2^ s^−1^) at 30 °C for 10 d. Ten-day-old plants were transferred to 0.5× MS liquid medium containing 100 μM salicylic acid, 100 μM IAA, 100 μM gibberellic acid, 100 μM methyl jasmonate, 10 mM 1-aminocyclopropane-1-carboxylic acid, or 100 μM ABA. Total RNA was extracted from leaves harvested at 12 h of treatment.

### 4.2. Determination of Photosynthetic Activity, Total Chlorophyll Content, and SPAD Value

To measure changes in photosynthetic activity, the middle section of the flag leaf of a plant grown in the paddy field under NLD conditions was adapted in the dark for 10 min. The *Fv*/*Fm* ratio was measured using an OS-30p+ instrument (Opti-Science, Hudson, NH, USA). To measure total chlorophyll content, detached leaves of plants grown in a growth chamber for 3 weeks were incubated in complete darkness or 50 μM ABA. Extracts obtained using 80% ice-cold acetone were centrifuged at 10,000× *g* for 10 min at 10 °C. The absorbance of the supernatants was measured at 647 and 663 nm. Chlorophyll content was calculated as previously described [73]. The change in SPAD value was measured in the flag leaves of plants grown in a paddy field under NLD conditions using a SPAD-502 instrument (Konica Minolta, Tokyo, Japan).

### 4.3. RT-qPCR Analysis

Total RNA was extracted from leaves using a Total RNA Extraction Kit (MGmed, Seoul, Korea) according to the manufacturer’s instructions. To synthesize first-strand cDNA, 2 µL of total RNA was subjected to reverse transcription (RT) in a 20 µL reaction volume using oligo(dT)_15_ primers and M-MLV reverse transcriptase (Promega, Madison, WI, USA) and diluted with 80 µl distilled water. qPCR was performed with gene-specific primers and normalized to rice *UBIQUITIN5* (*OsUBQ5*) (Os01g22490) (Table S1) according to the 2^−ΔΔ*C*t^ method [74]. The 20 µL reaction mixture included 2 µL of cDNA, 1 µL of 0.5 mM primers, and 10 µL of 2× GoTaq qPCR Master Mix (Promega). qPCR amplifications were performed in a LightCycler 480 (Roche, Basel, Switzerland) using the following conditions: 95 °C for 2 min followed by 40 cycles of 95 °C for 10 s and 60 °C for 1 min.

### 4.4. Plasmid Construction and Rice Transformation 

Full-length *ONAC096* cDNA was amplified by PCR using gene-specific primers listed in Table S1. The amplified fragments were ligated into the pCR8/GW/TOPO-TA cloning vector (Thermo Fisher Scientific, Waltham, MA, USA). The *ONAC096* cDNA was subsequently transferred into the pMDC32 gateway-compatible binary vectors using Gateway LR Clonase II Enzyme Mix (Invitrogen, Carlsbad, CA, USA), resulting in the pMDC32-ONAC096 construct. These plasmids were introduced into calli derived from Dongjin rice seeds via *Agrobacterium tumefaciens* (strain LBA4404)-mediated transformation [75]. *Agrobacterium*-infected calli were transferred to 0.5× MS solid medium containing 0.1 mg L^−1^ α-naphthaleneacetic acid (NAA) and 5 mg L^−1^ kinetin. Plantlets were regenerated from the callus under continuous light (90 µmol m^−2^ s^−1^) at 30 °C.

### 4.5. SDS-PAGE and Immunoblot Analysis

Total proteins were extracted from the flag leaves of plants grown in a paddy field under NLD conditions or from the detached leaves of 3-week-old plants incubated in complete darkness. Leaf tissue (10 mg) was homogenized in 100 µl of SDS sample buffer (50 mM Tris, pH 6.8, 2 mM EDTA, 10% (w/v) glycerol, 2% SDS, and 6% 2-mercaptoethanol), and 4 µL of the protein extract was separated by 12% SDS-PAGE (w/v) and transferred to an Immobilon-P Transfer Membrane (Millipore, Burlington, MA, USA). Antibodies against photosynthetic proteins (Lhca1, Lhcb1, PsaA, and PsbD) (Agrisera, Vännäs, Sweden) were used for immunoblot analyses. Horseradish peroxidase activity of secondary antibodies (Sigma, St. Louis, MO, USA) was detected using the ECL system (AbFRONTIER, Seoul, Korea) according to the manufacturer’s instructions.

## 5. Conclusions

We demonstrated that the NAC transcription factor ONAC096 participates in ABA-induced leaf senescence and tillering in rice (Figure 8). The downregulation of *ONAC096* delayed leaf yellowing and increased tiller number, leading to improved grain yields. Therefore, regulating the expression of *ONAC096* helps control panicle number and leaf senescence, representing a promising strategy for improving rice yields in the future.

## Supplementary Materials

Supplementary materials can be found at https://www.mdpi.com/1422-0067/20/20/5241/s1. Figure S1. Phylogenetic analysis of *Arabidopsis* NAC and rice NAC protein sequences. Figure S2. Expression profiles of rice NAC TFs throughout the growth period in the field. Figure S3. Amino acid sequence alignment of ONAC096 proteins. Figure S4. T-DNA insertion *onac096* mutants. Figure S5. Altered expression of CDGs and SAGs in *onac096* and *ONAC096-*OX plants. Table S1. Primers used in this study.

## Abbreviations

WT	wild type
NLD	natural long day
LD	long day
CDGs	chlorophyll degradation genes
SAGs	senescence-associated genes
DIS	dark-induced senescence
DDI	day(s) of dark incubation
DAS	day(s) after seeding
DAH	day(s) after heading
DT	day(s) after treatment
