# Supplementary material for: Mutation of ONAC096 Enhances Grain Yield by Increasing Panicle Number and Delaying Leaf Senescence during Grain Filling in Rice
Source: Int J Mol Sci. 2019 Oct 22;20(20):5241. doi: 10.3390/ijms20205241 (PMC6829889; doi:10.3390/ijms20205241)
Supplement: Supplementary file 1 [file ijms-20-05241-s001.pdf]

Supplementary Materials–Kang et al.

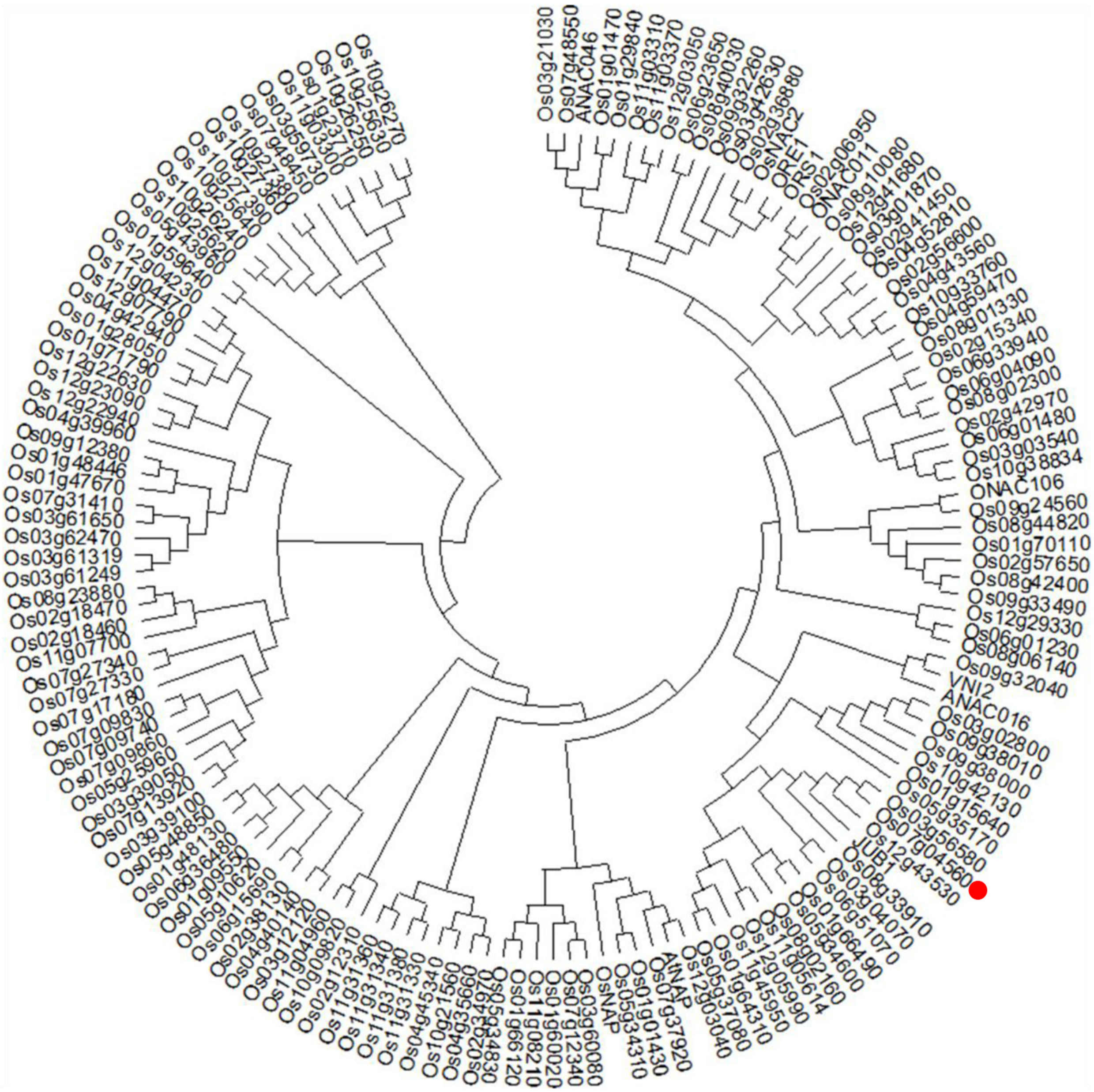

Figure S1. Phylogenetic analysis of *Arabidopsis* NAC and rice NAC protein sequences. The unrooted tree was generated using the ClustalX program with the neighbor-joining method. Bootstrap values from 1000 replicates are indicated at each node. The locus ID and names of *Arabidopsis* and rice NAC proteins were derived from the Rice Genome Annotation Project (http://rice.plantbiology.msu.edu/index.shtml) and various reports (OsNAP, Liang et al. 2014; ONAC106, Sakuraba et al. 2015; OsNAC2, Mao et al. 2017; ONAC011, El Mannai et al. 2017; Oresara1 (ORE1), Kim et al. 2009; Oresara1 sister1 (ORS1), Balazadeh et al. 2011; Jungbrunnen1 (JUB1), Wu et al. 2012; *Arabidopsis* NAC-like, activated by apetala3/pistillata (AtNAP), Guo et al. 2006; Vascular-related NAC-domain interacting (VNI2), Yang et al. 2011; ANAC016, Kim et al. 2013; ANAC046, Oda-Yamanizo et al. 2016). The red circle indicates ONAC096.

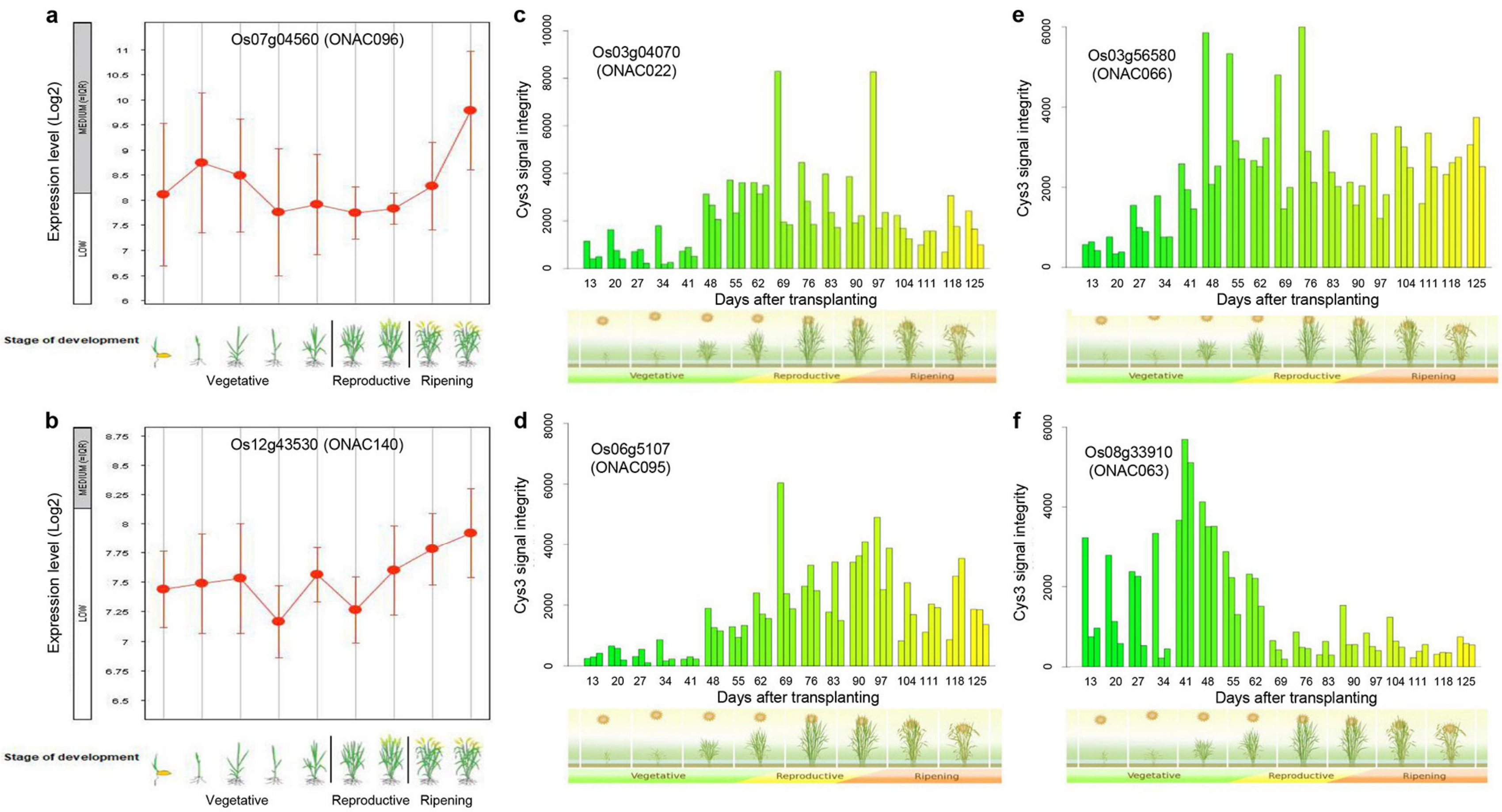

Figure S2. Expression profiles of rice NAC TFs throughout the growth period in the field. The data were obtained from GENEVESTIGATOR (https://genevestigator.com/gv/) (**a**,**b**) and RiceXPro (http://ricexpro.dna.affrc.go.jp/) (**c**–**f**).

```
ONAC096                    1 ---------MKR--GCEDELG-AGDV--ILRGVEEVEEEDDDDLVLPGFRFHPTDEELVT
Arabidopsis thaliana       1 --------------------M-------SGEGNLGKDHEEENEAPLPGFRFHPTDEELLG
Brachypodium distachyon    1 MVTYKEERDQNQSIAMEKVKN-NGEV----------VLGEEDDAALPGYRFHPTDEELVT
Sorghum bicolor            1 ---------MAKEIVMAGEHG-EGE------EVVLVEDEEEEEDMLPGFRFHPTDEELVT
Populus trichocarpa        1 -------------MVEKIINMNSQDHL-RSTNY--KDDDDEEEVQLPGFRFHPTDEELVG
Glycine max                1 --------------------MGVNEDFNQDIDYDHHEYVDDDDVPLPGFRFHPTDEELVS
Solanum lycopersicum       1 ---------------MENTIV-------CESKIKNSDDDEENDLLLPGFRFHPTDEELVG
Gossypium hirsutum         1 ---------MYLKVVKEIVRR-------IEMEVMEKMISEDEDVLLPGFRFHPTDEELVG

ONAC096                   47 FYLRRKIAEKRLSIEIIKEMDIYKHDPSDFLKTSTV--GSEKEWYFFCLRGRKYRNSIRP
Arabidopsis thaliana      34 YYLRRKVENKTIKLELIKQIDIYKYDPWDLPRVSSV---GEKEWYFFCMRGRKYRNSVRP
Brachypodium distachyon   50 FYLRRKVARKPLSIEVIREMDIYKHDPWDLPKASTV--GGEKEWYFFCLRGRKYRNSIRP
Sorghum bicolor           45 FYLRRKVAGKRLSIEIIKDFDIYKHDPWDLPKSSSI--LGEKEWYFFCLRGRKYRNSIRP
Populus trichocarpa       45 FYLRRMVDKKPLRIELIKQVEIYKYDPWDLPKSSCV---GDKEGYFFCKRGRKYRNSIRP
Glycine max               41 FYLQRKLDKKPISIELIKQIDIYKYDPWDLPKTSAT--GGEKEGYFFCRRGRKYRNSIRP
Solanum lycopersicum      39 FYLKRKVENKRIKLDLIKEVDIYKHDPWDLPMMGRV--GDNKEWYFFSMRGRKYKNSVRP
Gossypium hirsutum        45 FYLRRKVEKKLFSIDIIKHVDIYKHDPWDLPKVSKLSSGTEKEWYFFCRRGRKYRNSIRP

ONAC096                  105 NRVTGSGFWKATGIDRPICSAAGGGGGDCIGLKKSLVYYRGSAGKGTKTDWMMHEFRLPP
Arabidopsis thaliana      91 NRVTGSGFWKATGIDKPVYSN-----LDCVGLKKSLVYYLGSAGKGTKTDWMMHEFRLPS
Brachypodium distachyon  108 NRVTGSGFWKATGIDRPIYSAAAASSGESIGLKKSLVYYRGSAGKGTKTDWMMHEFRLPA
Sorghum bicolor          103 NRVTGSGFWKATGIDRPIHSAASGRAGDPIGLKKSLVFYRGSAGKGTKTEWMMHEFRLPP
Populus trichocarpa      102 NRVTGSGFWKATGIDKPVFSL-GGEGRDSIGLKKTLVYYRGSAGKGTKTDWMMHEFRLPT
Glycine max               99 NRVTGSGFWKATGIDKPVYSH-GGEGNDCIGLKKTLVYYRGSAGKGIKTDWMMHEFRLPS
Solanum lycopersicum      97 NRVTGSGFWKATGIDKPVHSQ-SN--ELCIGLKKSLVYYRGSAGKGTKTDWMMHEFRLPP
Gossypium hirsutum       105 NRVTGSGFWKATGIDKPIYSV-GGF-HDCIGLKKSLVYYKGSAGKGTKTDWMMHEFRLPP

ONAC096                  165 PPADDLAAGRSS--PPPSLQEAEVWTICRIFQRNITHKKQPQ-PQLAVAA--------AA
Arabidopsis thaliana     146 TTKTD-----------SPAQQAEVWTLCRIFKRVTSQRNPTILPPNRKPV----------
Brachypodium distachyon  168 AAA-------AN--ASPSMQEAEVWTICRIFRRNITYRKQQT-WRPPPAA--------AV
Sorghum bicolor          163 RAES------AH--TSPSEQEAEVWTICRIFRRNFTYKKHPQ-QQQIAGSSKVSAAATAA
Populus trichocarpa      161 KDNSTSTATV---KAKISDQEAEVWTLCRIFKRNVSCRKYTPDLKQLSTT----------
Glycine max              158 NTDNNNTNLRSSKNYVDVPQEAEIWTLCRIFKRNVSQRKHTPDLKQISAK----------
Solanum lycopersicum     154 IWKTNTSNGQHLPNLKNIAAEAEVWTLCRIFKRISNYKRFTPDWKQQQPV----------
Gossypium hirsutum       163 PSTTTLSST-----NKDNLPEAEVWTLCRIFKRDVSSRKFASDWQNKKNK----------

ONAC096                  214 VPAPVPDATSSITGSLESDSAG-DD-VVEYM------NTLQPP-PASNVNGGY------S
Arabidopsis thaliana     185 -I------TLTDTCSKTSSLDSDHTSHRTVD-----SMSHEPPLPQP--QNPYWNQHI--
Brachypodium distachyon  210 STAVAADSSSNTAGSFESSDGGGDD-YMAQA------ATTGPPCIIPHVQQHHGNLQLGA
Sorghum bicolor          214 VVTTQPGESSSVTGSLESDTGD-E-----YT------NDLPQPTQAPAIVDGY------D
Populus trichocarpa      208 -PQQPPIDTSSKLCCQVESNYTQES-YVNFGAPLIQHYDNKPPVH-----------HVKE
Glycine max              208 -RQSI-HDKSSRMSNVEFNTTNQES-YINFGGHYHNEQ--KPTINYT---------NSDQ
Solanum lycopersicum     204 -VKQSFVDTSSKACSVQSEISDDQSNNVI-N-----FKKM----AFP--QKSIMNASCGG
Gossypium hirsutum       208 -QNTNTSAS SRACSVESENSLKVENFGVLD-----DKGIE---------RKLQNDHF—

ONAC096                  259 NQR-----YFQEQWNSSSNDNTTVFHQHAAAAPPPEPSPATAMAGFGHDQSVLSSPA---
Arabidopsis thaliana     229 -----------------VGFNQPTY-T----------GNDNNL------LMSFWNG--NG
Brachypodium distachyon  263 TNGGFFSQQFQGQWSSVPPPTLPLPDQKPL-------NPASAPIAFHLNDHSLA------
Sorghum bicolor          256 YDYGYGYDQQQGQWNSHA-----------LHAAATAPLPSPTMAAFHH--SVLSSPAAGG
Populus trichocarpa      255 -----RKPLHVDQL--------------------SYVAQPPSM------ASSLNI---SS
Glycine max              254 -----RNQYHVMTHHQLCA---PVAQQH------QQPQQLTSP------SSNFWI---NN
Solanum lycopersicum     251 -----NLNYQVDQRNSYYNNSQLITTMP------DQ-SPFTSS------NSIFWNT--RA
Gossypium hirsutum       251 -----------------LGGNQLFAAT----------TQVLPS------YLSFSNP—NA-

ONAC096                  311 -PSDFYYKDGC--NDDIYRMVMELADPSLFYDHIYA-----
Arabidopsis thaliana     253 --GDFIGDS--ASWDELRSVIDGNTKP--------------
Brachypodium distachyon  310 AASDLYKVDGY--LEEIAR-MMEVTDPHPAAIYDYRYA---
Sorghum bicolor          303 GLDDMYYKDGSSSWDDIGRMVMELTDPSPGVFSFYDTRSYV
Populus trichocarpa      281 PYGNQILTH--GDWDELTSVVDCAFDPFLV-----------
Glycine max              291 PPGNDFFTF--DNWDELGSLVKFAVDSPSL-----------
Solanum lycopersicum     291 EDQEYLFSH--GNWDELKSVVDLAIDPRSLFGFK-------
Gossypium hirsutum       276 --ADEYFGQ--EHWDELRPVVDYTIAKSLQYSDW-------
```

Figure S3. Amino acid sequence alignment of ONAC096 proteins. The amino acid sequences of ONAC096 from rice and other plant species homologous to ONAC096 were obtained from NCBI through BLAST analysis. Sequence alignment was performed using ClustalW with default parameters. The sequences are as follows: *Arabidopsis*, XP_015646514, *Brachypodium distachyon*, XP_003559373; *Sorghum bicolor*, XP_002459331; *Populus trichocarpa*, XP_024461145; *Glycine max*, NP_001241333; *Solanum lycopersicum*, XP_004243883; *Gossypium hirsutum*, XP_016753414. The black bars represent the conserved NAM domains of NAC proteins.

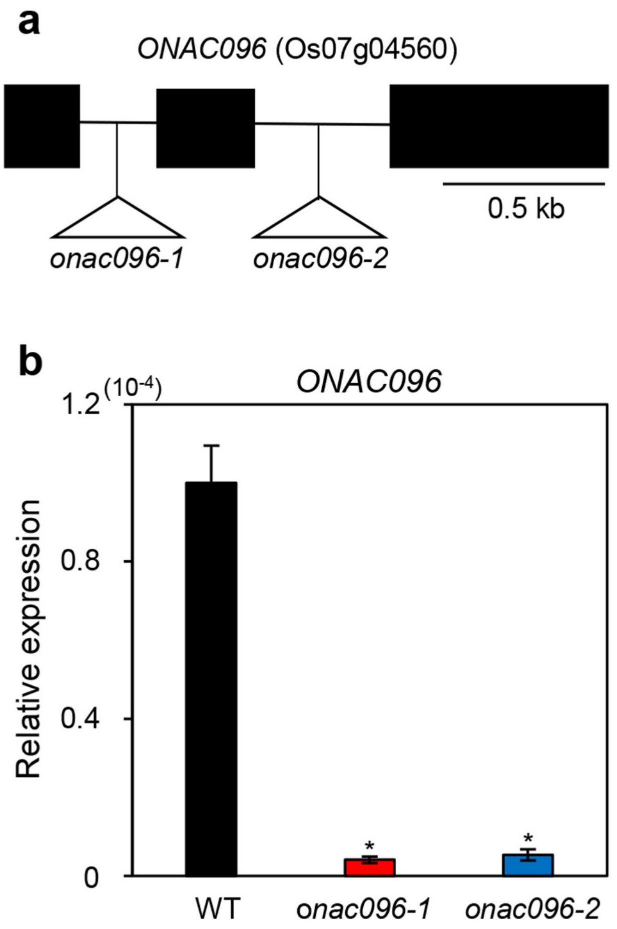

Figure S4. T-DNA insertion *onac096* mutants. (**a**) Schematic diagram of the positions of the T-DNA insertion in two independent *onac096* mutants. The black boxes and lines represent exons and introns, respectively. The white triangles indicate the positions of T-DNA insertions (*n096-1*, PFG_1B-02928 and *n096-2*, PFG_3A-08770). (**b**) *ONAC096* expression in wild-type (WT) and *onac096* plants. Total RNA was isolated from the leaves of 3-week-old plants grown in paddy soil under long-day conditions. *ONAC096* transcript levels were measured by RT-qPCR and normalized to that of *OsUBQ5*. Relative expression was calculated using the ΔΔCT method. Asterisks indicate statistically significant differences between *onac096* and the WT, as determined by Student's *t*-test (*$p < 0.05$). The experiments were repeated twice with similar results.

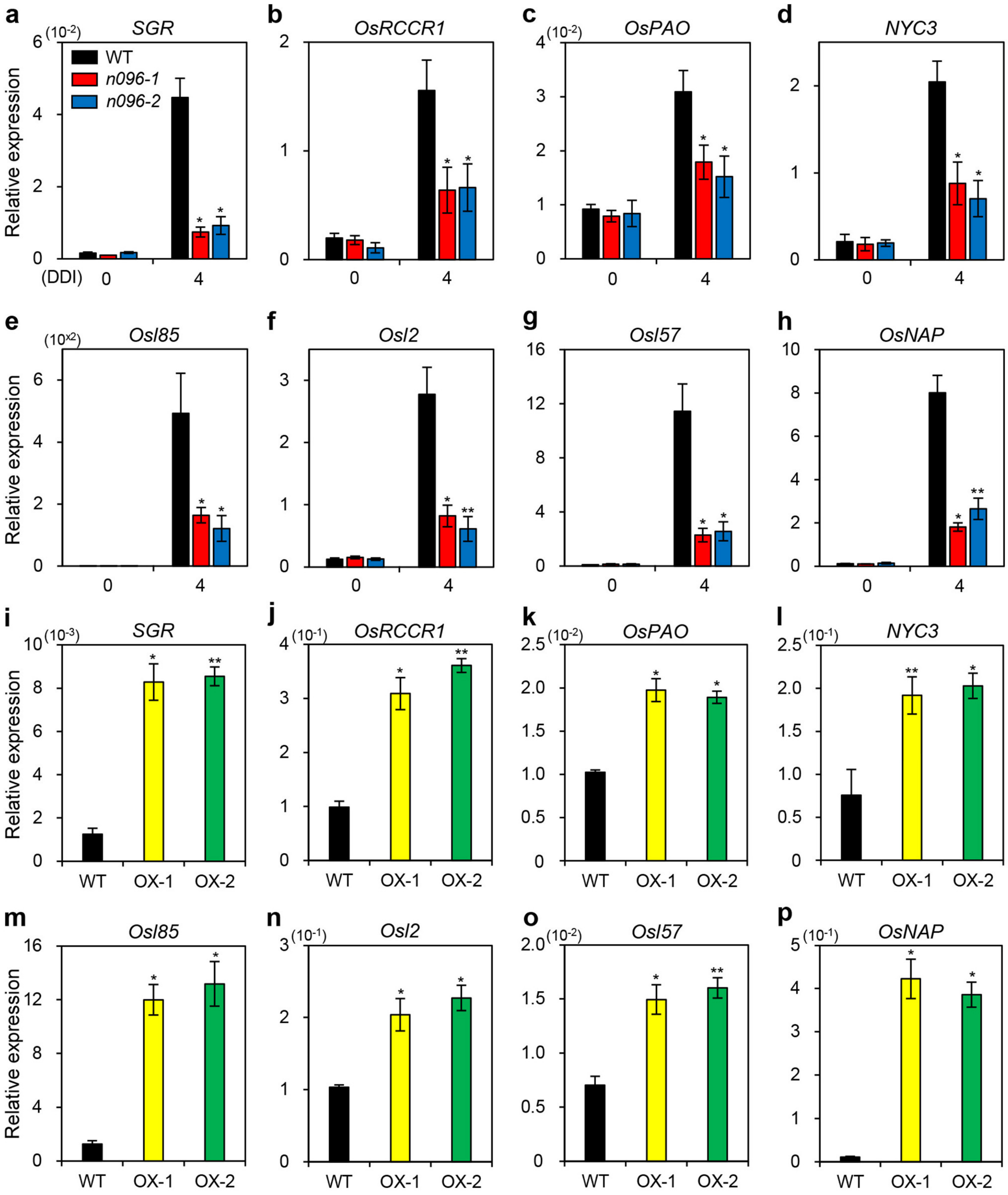

Figure S5. Altered expression of CDGs and SAGs in *onac096* and *ONAC096*-OX plants. Total RNA was isolated from the detached leaves of 3-week-old WT and *onac096* plants (*n096-1* and *n096-2*) incubated in 3 mM MES buffer (pH 5.8) in complete darkness at 28 °C (**a**–**h**) under long-day conditions (14.5 h light per day) or the attached leaves of WT and *ONAC096*-OX plants (OX-1 and OX-2) grown in paddy soil. for 3 weeks under NLD conditions (**i**–**p**). The transcript levels of chlorophyll degradation genes (CDGs; **a**–**d, i**–**l**) and senescence-associated genes (SAGs; **e**–**h, m**–**p**) were measured by RT-qPCR and normalized to that of

OsUBQ5. Relative expression was calculated using the ΔΔCT method. Mean and standard deviations were obtained from three biological repeats. Asterisks indicate statistically significant differences between *onac096* mutants and *ONAC096*-OX compared to the WT according to Student's *t*-test (*$p < 0.05$ and ** $p < 0.01$). The experiments were repeated twice with similar results. DDI, day(s) of dark incubation.

Table S1. Primers used in this study.

**A. Primers for verification of T-DNA insertion**		
**Primer names**	**Left primers (5′ → 3′)**	**Right primers (5′ → 3′)**
PFG_1B-02928	CATTAAAGCTGGACCAGATGG	AACCACTTGCGGATTAATGC
PFG_3A-08770	TTGGATGCCTGATTAAGGTTG	CCGTTCTTGCACATTCACAC
pGA2715	CTAGAGTCGAGAATTCAGTACA	TTGGGGTTTCTACAGGACGTAAC
B. Primers for RT-qPCR		
Genes	Forward primers (5′ → 3′)	Reverse primers (5′ → 3′)
ONAC096	CAAGCATGATCCCTCCGACT	TCCCTCGAAGGCAGAAGAAG
SGR	AGGGGTGGTACAACAAGCTG	GCTCCTTGCGGAAGATGTAG
NYC3	TGTCGTTGCCATGTGAAGAT	TTGGTCACGCCACAAATCTA
OsPAO	GGAAATCCTAGCCAAGAAGTGTTG	CGCAGGAATCCCAGCAGTT
OsRCCR1	CGCATTTCCTCATGGAATTT	CTTCTCACGCTGTTTGTCCA
Osl2	CGCAGACAACAAATCGCCAA	CTCCAGCAACTCTAACCAGCA
Osl85	GAGCAACGGCGTGGAGA	GCGGCGGTAGAGGAGATG
Osl57	ACCCTAAAGTAAATGAAGTC	CCTGCTCTTGTCTTGTTA
OsNAP	CAAGAAGCCGAACGGTTC	GTTAGAGTGGAGCAGCAT
OsCKX2	CCGGGATAGCCTACAAGCAG	CCAGAGATTGGCACCGAAGT
OsPIN2	GAGGTACGACTTCCATGGGC	TTGAGGTACGGCTTGGACAC
OsTB1	CCCAGCTTGAAGCTTTTGCT	ACAACACTGCAACTATCCCTATCACT
OsPIN5b	GGGCTTCATGCCGATGTACT	TAGACAAAGCCCAGAACCGC
OsTIR	TCCAGGTGCTCCGCCTCGTCTCCT	CCGGGAAGAGGCTGAGCCAATGAA
OsABI5	CGAAGCTGAACTGAACTATC	CTGGCTGCCACCCCTATTTG
OsEEL	GCAGAAGCGCATGATCAAGA	GACGCAGCTAGGGAATGTTG
OsUBQ5	ACCACTTCGACCGCCACTACT	ACGCCTAAGCCTGCTGGTT
C. Primers for plant transformation		
Primer names	Forward primers (5′ → 3′)	Reverse primers (5′ → 3′)
ONAC096	ATGAAGAGGGGTTGTGAAGATGA	TTAAGCATATATATGATCATAGAAC
35S promoter	CTATCCTTCGCAAGACCCTTC	
